# The spectrum of copy number variation in the Pan-Canadian HostSeq databank

**DOI:** 10.1371/journal.pone.0359210

**Published:** 2026-09-25

**Authors:** Navneet Aujla, Bhooma Thiruvahindrapuram, Selina Casalino, Erika Frangione, Radhika Mahajan, David Di Iorio, Chun Yiu Jordan Fung, Lochana Jayachandran, Georgia MacDonald, Gregory Morgan, Dawit Wolday, Juliet Young, Maahil Arshad, Marc Clausen, Saranya Arnoldo, Alexandra Binnie, Bjug Borgundvaag, Sunakshi Chowdhary, Marc Dagher, Luke Devine, Steven Marc Friedman, Anne-Claude Gingras, Lee W. Goneau, Zeeshan Khan, Elisa Lapadula, Tony Mazzulli, Allison McGeer, Shelley McLeod, Chloe Mighton, Trevor J. Pugh, David Richardson, Jared Simpson, Seth Stern, Ahmed Taher, Lisa Strug, Yvonne Bombard, Hanna Faghfoury, Elena Greenfeld, CGEn HostSeq Initiative, Stephen W. Scherer, Jennifer Taher, Abdul Noor, Jordan Lerner-Ellis

**Affiliations:** 1 Mount Sinai Hospital, Sinai Health, Toronto, Ontario, Canada; 2 Lunenfeld-Tanenbaum Research Institute, Sinai Health, Toronto, Ontario, Canada; 3 The Centre for Applied Genomics and the Program in Genetics and Genome Biology, The Hospital for Sick Children, Toronto, Ontario, Canada; 4 University of Toronto, Toronto, Ontario, Canada; 5 Unity Health Toronto, Toronto, Ontario, Canada; 6 William Osler Health System, Brampton, Ontario, Canada; 7 Schwartz/Reisman Emergency Medicine Institute, Sinai Health, Toronto, Ontario, Canada; 8 University Health Network, Toronto, Ontario, Canada; 9 Women’s College Hospital, Toronto, Ontario, Canada; 10 Dynacare Medical Laboratories, Brampton, Ontario, Canada; 11 Mackenzie Health, Richmond Hill, Ontario, Canada; 12 Ontario Institute for Cancer Research, Toronto, Ontario, Canada; 13 McLaughlin Centre and Department of Molecular Genetics, University of Toronto, Toronto, Ontario, Canada; West Bengal University of Animal and Fishery Sciences, INDIA

## Abstract

The integration of copy number variant (CNV) workflows into genome sequencing (GS) analysis pipelines allows for the identification of CNVs implicated in disease. Here, we generated a novel resource of CNVs identified in the HostSeq population cohort from Canada, identified the prevalence of recurrent CNVs associated with neurodevelopmental disorders, and determined CNVs of potential clinical relevance for reproductive planning and personal disease risk. GS data and CNV calls were generated for 10,488 participants from across Canada as part of the HostSeq initiative. The CNV calls were filtered to generate a rare dataset, which was further filtered into the OMIM morbid, ClinGen dosage, and DECIPHER datasets. CNV deletions were stratified into either Tier 1, 2, or 3 based on the inheritance pattern of the genes involved. A putatively pathogenic dataset was generated by identifying CNVs in the ClinGen dosage and DECIPHER datasets with at least 80% overlap with previously identified pathogenic CNVs. A total of 8,543,334 CNV calls were generated. Filtering for rare variants yielded 36,631 CNVs, of which 9,922 (27.08%) encompassed at least one OMIM gene, 728 (1.99%) had at least 10% overlap with a DECIPHER region, and 1,136 (3.10%) encompassed at least one ClinGen dosage-sensitive gene. CNV deletions were stratified into 1,833 Tier 1 deletions, 32 Tier 2 deletions, and 184 Tier 3 deletions. There were 82 CNVs identified in regions associated with neurodevelopmental disorders. Of the genes with either a ClinGen dosage sensitivity score or overlap with a DECIPHER region, 206 CNVs were deemed as putatively pathogenic. We were able to detect a wide range of CNVs, highlighting the use of integrating CNV workflows into the analysis pipeline, and generated a data resource for medical genomics for the Canadian population encompassing the full spectrum of CNVs.

## Introduction

Understanding the spectrum of human genetic variation is essential to determining the relationship between variants and genetic disease. One such type of genetic variation is copy number variants (CNVs). CNVs are defined as deletions or duplications of genomic regions greater than 1 kilobase (kb), and are estimated to cover approximately 4.8% to 9.5% of the human genome [1–3]. CNVs can span many genes and have been implicated as disease-causing in numerous inherited conditions, including multiple forms of hereditary cancer, and alpha-thalassemia [4,5]. In some instances, CNVs are the predominant or sole known cause of disease for a condition and therefore play an important role in understanding how genetic changes impact human health.

With the advent of next-generation sequencing (NGS), novel tools to detect CNVs from sequencing data have been developed, making it easier to incorporate CNV workflows into standard bioinformatics analysis pipelines [6–8]. To understand the association between CNVs and hereditary disease, one must first appreciate the broad spectrum of CNVs present in healthy individuals. Numerous large-scale studies have analyzed CNV variation in different ancestral groups, with the goal of either diagnosing specific disease(s), or identifying the frequency of CNVs within healthy cohorts [4,9–12]. Moreover, the spectrum of CNVs associated with carrier status, or inherited conditions with reduced disease penetrance or variable expressivity are often not evaluated. CNV datasets relevant to the Canadian population face the same limitations of focusing primarily on healthy controls and/or affected individuals [13–21].

In this study, we present CNV data from 10,488 Canadians who underwent genome sequencing (GS) through the HostSeq initiative [22,23]. HostSeq is a Canadian database of GS and health-related data from several clinical and epidemiological studies across Canada [22,23]. The HostSeq cohort is a valuable resource for genomic analysis, particularly for CNV analysis, as the data is derived from 30X GS. Additionally, participants were enrolled based on a COVID-19 diagnosis, rather than on the absence or presence of any clinical indications or underlying disease etiology. These participants represent a range of genetic ancestries across Canada, including those currently underrepresented in typical genomic datasets, such as Canadian indigenous populations. Furthermore, the Government of Canada estimates that approximately 38.7% of Canadian adults tested positive for or suspected a COVID-19 infection in the summer of 2022, and nearly doubled to 64.4% by the summer of 2023 [24]. Therefore, we presume that the CNVs in the HostSeq cohort are representative of the CNV spectrum in the general, ostensibly healthy Canadian population. Using stringent filtering criteria, the cohort can be screened for clinically significant CNVs, which can be useful in determining disease risk or carrier frequencies for rare conditions. This data allows us to determine the population frequency of clinically significant CNVs, as well as CNVs identified as risk factors for conditions such as neurodevelopmental disorders. This information will be a valuable resource for genetic counseling. In this study, we 1) present an overview of the spectrum of CNVs identified in the HostSeq cohort, 2) identify the prevalence of recurrent CNVs associated with neurodevelopmental disorders, and 3) identify CNVs of potential clinical relevance for reproductive planning and personal disease risk(s).

## Methods

### Ethics statement

This study was approved by the Research Ethics Board of Mount Sinai Hospital (CTO Project ID: 3302; Sponsor Study ID: 424901). Written or verbal informed consent was obtained from all participants, or their parents/guardians/substitute decision makers, prior to inclusion in the study. Verbal consent was documented, dated, and signed by the person obtaining consent, following REB approved protocols.

### Study enrollment

10,645 participants were enrolled in one of the various studies that were a part of the HostSeq consortium [22,23]. Samples were collected from participants across Canada and sequenced at one of three sites: The Center for Applied Genomics (TCAG; Hospital for Sick Children, Toronto, ON), McGill Genome Centre (McGill University, Montreal, QB), or Canada’s Michael Smith Genome Sciences Centre (Vancouver, BC). All samples were sequenced on the Illumina NovaSeq6000 at 30X coverage. The samples were aligned to the Genome Reference Consortium human build 38 (GRCh38 assembly version GCA_000001405.15) and processed using Illumina DRAGEN software v3.8.4 at the TCAG [25]. The authors did not have access to any data that could be used to identify any participants.

### CNV calling

CNV calls were generated for each sample in May 2025 at the TCAG using Illumina DRAGEN software v3.8.4, with single sample self-normalization [25]. Default values were used for all parameters except *–cnv-filter-length*, which was decreased to 5kb. Sample QC was done by computing the percentage of each chromosome that is a CNV and the number of CNV calls made for each sample. Samples were excluded if either of these metrics was greater than 3 standard deviations above the mean, resulting in a final total of 10,488 participants. Batch and sequencing-site assessment were not done. Annotated CNVs were received directly from the TCAG.

### CNV filtration

The CNV calls for each participant were accessed on May 6^th^ 2025 and were filtered for inclusion based on CNVs spanning the exonic region of any gene. CNVs with either greater than 90% overlap with regions with gaps and/or segmental duplications were filtered out. Additionally, CNVs below a size threshold of 15kb and those that did not pass all sample filters were excluded to reduce the number of false calls. The filtered CNV calls for all participants were merged to generate a preliminary dataset. To reduce the number of sequencing artifacts, the internal population frequency per variant was calculated using variants with at least 50% reciprocal overlap, matched by variant type. Variants observed at a frequency of greater than 1% were excluded, generating the rare variant dataset. The rare variant dataset was used for further filtration and analysis. The number of unique CNVs in the rare dataset and all subsequent datasets was determined by counting deletions and duplications with unique breakpoints. CNVs in problematic regions with near-identical breakpoints were counted as unique CNV calls.

### Prevalence estimates of CNVs per gene

The prevalence of deletions, duplications, and all CNVs per 10,000 individuals was estimated for each gene using a formula of $\frac{alleleCount}{totalParticipants}*\text{1}\text{0},\text{0}\text{0}\text{0}$. The annotated CNVs did not contain gene information for any CNVs spanning 100 genes or more, preventing allele from being determined from the rare dataset alone. To overcome this, the allele count for each unique CNV was first calculated considering the sex and copy number of all participants carrying that CNV. To determine the genes in each CNV, the MANE transcript track (dated 2025-12-08) from the UCSC Genome browser was downloaded. The CNV counts were overlapped with the MANE coordinates using the GenomicRanges v.1.54.1 package in R, and the allele count per gene was determined by summing the allele counts of all CNVs that overlapped a given gene [26,27]. A 95% confidence interval (C.I.) for the prevalence estimates was determined using the Wilson Score interval test, without a Yates’ continuity correction. All statistical analyses were conducted in R v.4.3.1 (S1 File) [27].

### Tier categorization structure of recessive genes

All deletions from the rare CNV dataset were stratified by the inheritance pattern of the conditions associated with the genes deleted, using the OMIM phenotype and associated inheritance pattern for each gene that was included in the annotated file [28]. CNVs were stratified into either Tier 1, 2, or 3. Tier 1 deletions involved genes with only a recessive inheritance pattern. Tier 2 deletions encompassed at least one gene with only a recessive inheritance pattern and at least one gene with both recessive and dominant inheritance patterns. Tier 3 deletions encompassed only genes with both recessive and dominant inheritance patterns [29]. Any CNV encompassing a gene with only dominant inheritance patterns was excluded, based on the methodology presented by Boone et al. [29]. For the purposes of stratification, “recessive” encompassed autosomal recessive (AR), X-linked recessive (XLR) and X-Linked (XL) inheritance patterns, while “dominant” encompassed autosomal dominant (AD) and X-linked dominant (XLD) inheritance patterns. Genes with only provisional gene-disease relationships, those associated with non-disease conditions, and genes associated with susceptibility to disease did not contribute to the stratification of the CNVs in which they were identified.

### Preliminary curation of previously identified pathogenic CNVs

The rare variant dataset was further subdivided into the OMIM morbid dataset, ClinGen dosage dataset, and DECIPHER dataset based on 1) the presence of OMIM morbid genes in the CNV, 2) a known ClinGen Dosage sensitivity score for any gene contained within the CNV, or 3) a greater than 10% overlap with a known DECIPHER region [28,30,31]. These filters were not mutually exclusive, so if a CNV contained a ClinGen dosage-sensitive gene, an OMIM gene, and had more than 10% overlap with DECIPHER, it would have been captured by all three filters. The rare CNV dataset was also filtered to identify the frequency of previously published recurrent CNVs associated with neuropsychiatric phenotypes [32].

The ClinGen dosage and DECIPHER datasets were manually reviewed to identify any CNVs overlapping with previously identified pathogenic CNVs in internal databases. At the time of analysis, the pathogenic regions were only available for hg19, so CNV calls were converted from hg38 to hg19 using the University of California, Santa Cruz (UCSC) Batch Coordinate Conversion (Liftover) tool [33]. CNV calls with at least 80% overlap were flagged as putatively pathogenic; they were not subjected to formal assessment.

### Data visualization

Figures were generated in R v.4.3.1 using the circlize v. 0.4.16, ggplot2 v.3.5.1, and cowplot v.1.2.0 packages [27,34–36].

## Results

### Aim 1

#### Overview of CNVs.

**Fig 1:**
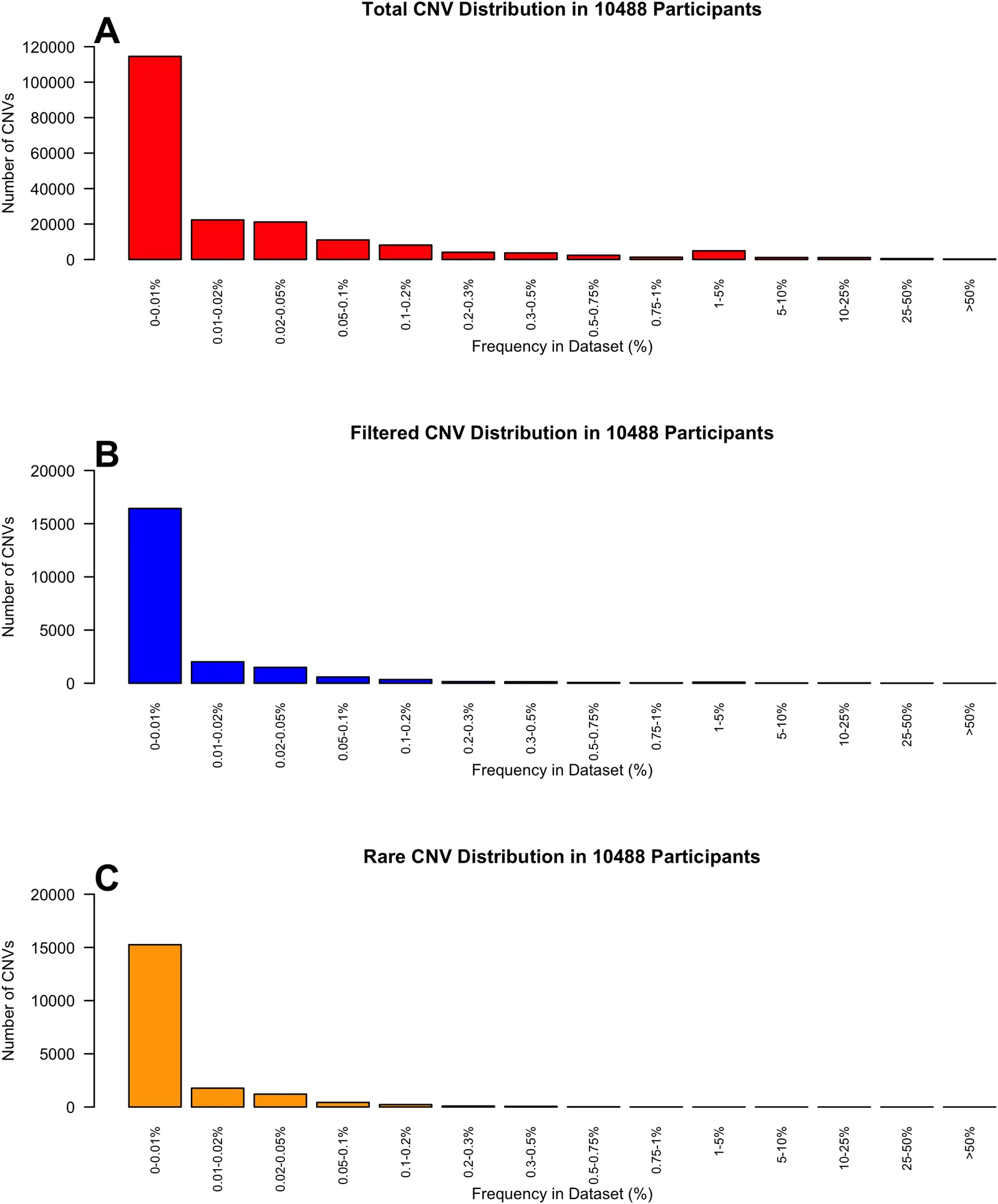
Distribution of CNVs in the HostSeq cohort. (A) Distribution of the frequency of all 8,543,334 CNVs in HostSeq. (B) Distribution of the frequency of the 226,318 CNVs in the filtered dataset. (C). Distribution of the frequency of the 36,631 CNVs in the rare dataset.

**Fig 2:**
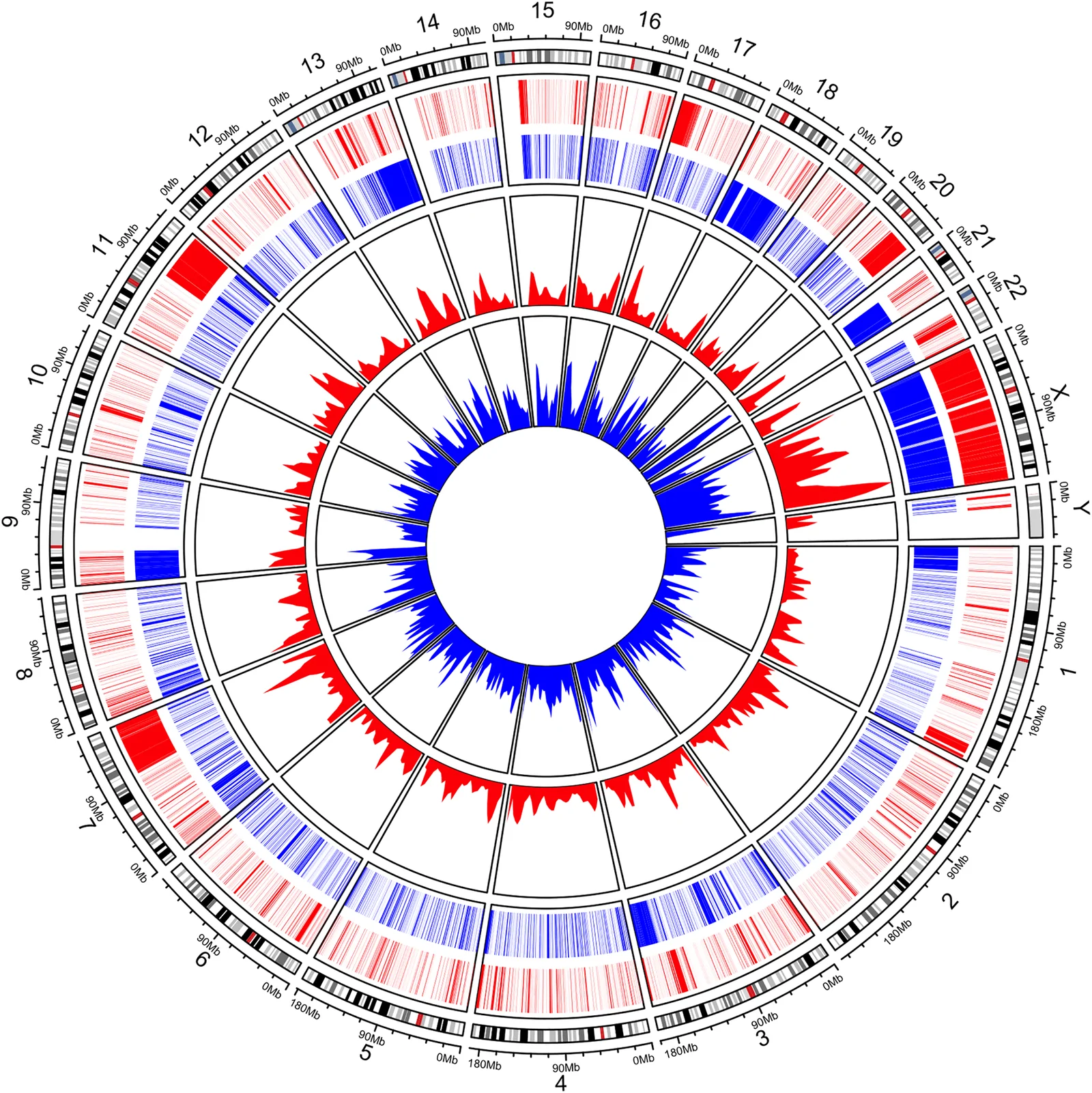
Circos plot of rare CNVs identified in the HostSeq cohort. The outer track depicts the distribution of deletions (red) and duplications (blue) along each autosomal chromosome. The two innermost tracks are the density of deletions (red) and duplications (blue). The higher the peak, the more CNVs were identified within that area.

A total of 8,543,334 CNVs were observed in 10,488 participants before filtration (S1 Table). On average, each participant had 670 deletions and 144 duplications. Removing duplicate CNV calls yielded 196,614 CNVs with unique breakpoints, 114,557 (58.26%) of which were unique to each participant. A total of 2,975 CNVs were seen in over 5% of the HostSeq participants. After preliminary filtration, 226,318 CNVs remained, a reduction of 97.35% (Fig 1, S2 Table). 172,864 (76.38% of 226,318 filtered CNVs) of the filtered calls were attributed to 183 variants, the most frequent of which were a duplication of chr12:21355–60453 and a deletion of chr16:15029974, which were observed in 10,450 and 8,912 participants, respectively (S2 Table). The chromosome 12 duplication contained the genes *WASH8P* and *FAM138D*, and the chromosome 16 deletion contained the genes *PDXDC1* and *NTAN1*. None of these genes has any known disease associations in OMIM or ClinGen [28,30]. These two CNVs spanned known problematic regions in the UCSC Genome Browser, and were not observed at similar levels in gnomAD SV v.4.1.0, nor in the DGV, suggesting that they were artifacts of sequencing [37–39]. The subsequent filtration for rare variants removed these 183 variants along with 2,203 other variants. After filtration, only 36,631 rare CNVs remained (0.42% of all 8,543,334 CNVs and 16.19% of 226,318 filtered CNVs). Removing duplicated CNV calls reduced the total count to 19,081 CNVs (52.09% of 36,631 rare CNVs) with unique break points, the vast majority of which were unique to each participant (15,256 or 79.96% of 19,081 unique CNV calls, and 41.64% of 36,631 rare CNVs) (Figs 1 and 2, S3–S4 Table). The remaining 3,825 variants were observed at a frequency of less than 1% in the entire cohort, with the ten most common variants listed in Table 1. The prevalence of CNVs per 10,000 individuals was also calculated for each gene and ranged from 0 (95% C.I.: 0.00, 3.66) to 228.83 (95% C.I.: 201.91, 259.25) for deletions and duplications (S5 Table).

**Table 1:** Ten most common rare CNVs seen in HostSeq Cohort.

Chromosome	Start (bp)	Stop (bp)	Variant Type	Number of Participants (n = 10,488)	Percent of Participants (%)
chr1	206008265	206024856	DEL	98	0.93
chr7	112301403	112322876	DEL	93	0.89
chr18	1902604	1984556	DEL	92	0.88
chr12	7844086	7971371	DEL	90	0.86
chr14	98711316	98727041	DEL	86	0.82
chr2	184381486	184399766	DEL	85	0.81
chr9	69483611	69505676	DUP	82	0.78
chr16	27324412	27339743	DEL	73	0.70
chr8	8234481	8290123	DUP	73	0.70
chr5	112928246	112944020	DEL	70	0.67

#### Tiered analysis of recessive deletions.

**Fig 3:**
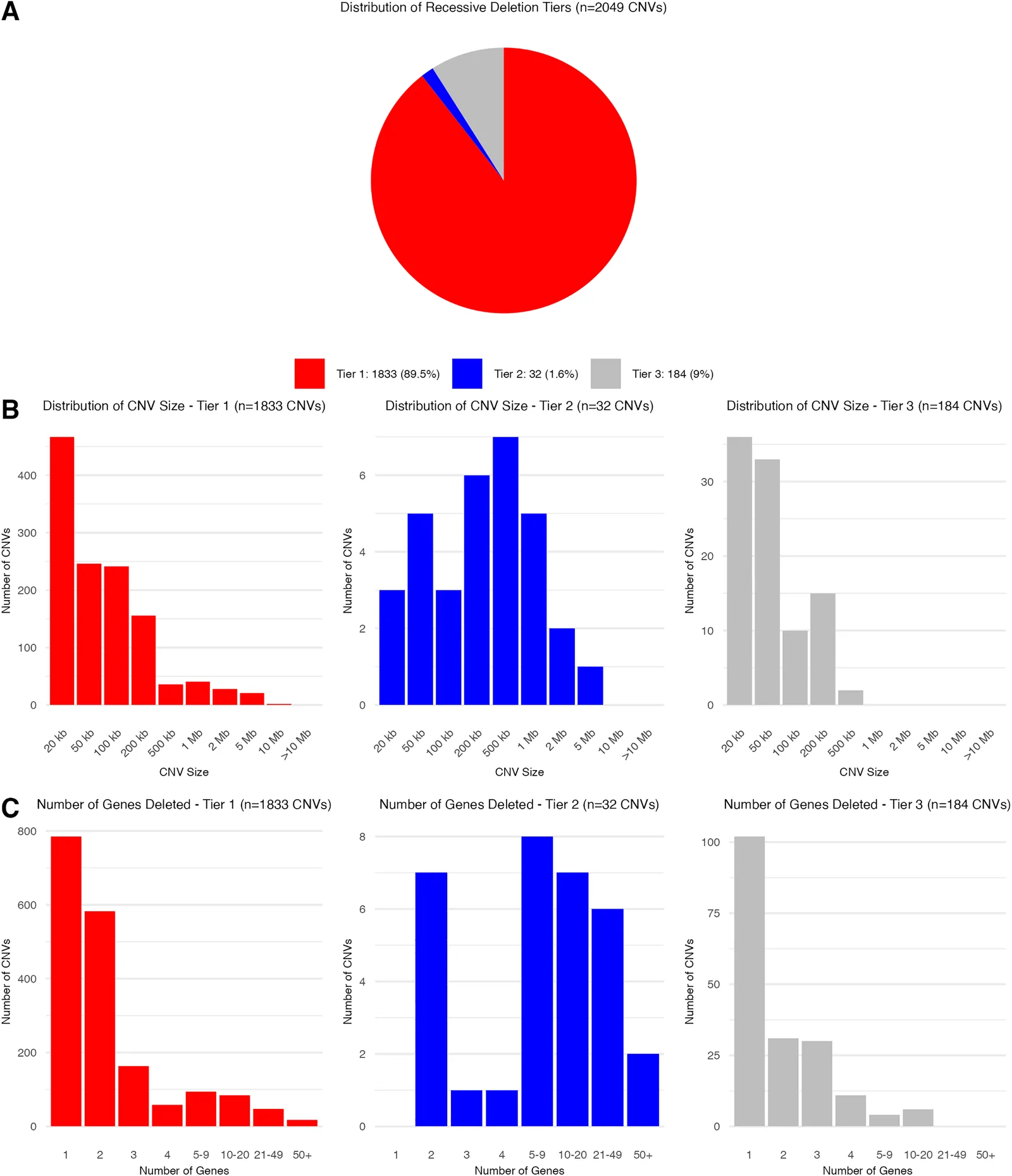
Tier 1, 2, and 3 Deletions in the HostSeq Cohort. (A) The distribution of deletions among the three tiers is coloured in red, blue, and grey for Tiers 1, 2, and 3, respectively. (B) Histograms showing the distribution of CNV size for each tier. (C) Histograms showing the number of genes deleted in each tier.

A total of 9,922 CNVs (27.08% of 36,631 rare CNVs) encompassing at least one OMIM morbid gene were identified, 3,645 of which were deletions (Fig 3, S6 Table). Deletions were categorized into Tier 1, 2, and 3 based on the genes’ inheritance pattern [29]. Tier 1 deletions involved genes with only a recessive inheritance pattern. Tier 2 deletions encompassed at least one gene with only a recessive inheritance pattern and at least one gene with both recessive and dominant inheritance patterns. Tier 3 deletions encompassed only genes with both recessive and dominant inheritance patterns [29]. A total of 1,596 deletions were excluded from analysis. Of the remaining 2,049 CNVs, the majority were categorized as Tier 1 (1,833 CNVs or 89.45% of 2,049 CNVs), followed by Tier 3 (184 CNVs), and lastly Tier 2 (32 CNVs) (Figs 2 and 3, S7 Table). The most frequent deletion was observed in 48 (0.46%) individuals and involved *NPHP1*, which is associated with Joubert Syndrome 4 (OMIM: 609583), Nephronophthisis 1 (OMIM: 256100), and Senior-Loken Syndrome 1 (OMIM: 266900).

### Aim 2

There were 82 CNVs identified overlapping pathogenic variants linked to neurodevelopmental phenotypes (Table 2, S8 Table) [32].

**Table 2:** Frequency and prevalence estimates of known recurrent pathogenic CNVs in the HostSeq cohort.

Region	Type	Count of Participants (n = 10,488)	Frequency (%)	Prevalence (95% C.I.) per 10,000
22q11.21 proximal (*TBX1*)	DUP	13	0.12	12.40 (7.25, 21.20)
17q12 (*HNF1B*)	DUP	5	0.05	4.77 (2.04, 11.16)
17q12 (*HNF1B*)	DEL	0	0	0.00 (0.00, 3.66)
15q11.2 (*NIPA1*)	DEL	29	0.28	27.65 (19.26, 39.68)
16p11.2 proximal (*TBX6)*	DEL	3	0.03	2.86 (0.97, 8.41)
16p11.2 proximal (*TBX6*)	DUP	7	0.07	6.67 (3.23, 13.77)
16p11.2 distal (*SH2B1*)	DEL	2	0.02	1.91 (0.52, 6.95)
16p11.2 distal (*SH2B1*)	DUP	4	0.04	3.81 (1.48, 9.80)
16p12.1 proximal (*CDR2*)	DEL	9	0.09	8.58 (4.52, 16.30)
16p13.11 (*MYH11*)	DEL	4	0.04	3.81 (1.48, 9.80)
1q21.1 distal (*GJA5*)	DEL	2	0.02	1.91 (0.52, 6.95)
1q21.1 distal (*GJA5*)	DUP	0	0	0.00 (0.00, 3.66)
1q21.1 proximal (*RBM8A*)	DUP	4	0.04	3.81 (1.48, 9.80)

### Aim 3

The rare dataset was further filtered using ClinGen dosage scores. A ClinGen dosage score of 3 indicates sufficient evidence for haploinsufficiency or triplosensitivity. A haploinsufficiency score of 30 corresponds to a gene with a condition that is inherited in an autosomal recessive manner, or carrier status in the case of heterozygotes [40,41]. Filtration yielded 220 (0.60% of 36,631 rare CNVs) deletions with a haploinsufficiency score of 3, eight (0.02% of 36,631 rare CNVs) duplications with a triplosensitivity score of 3, and 908 (2.48% of 36,631 rare CNVs) deletions with a ClinGen haploinsufficiency score of 30, six of which were in the homozygous state (S9–S11 Tables).

A total of 728 CNVs (1.99% of 36,631 rare CNVs) that had at least 10% overlap with DECIPHER regions were identified (S12 Table) [31]. CNVs with a ClinGen dosage score, and those with greater than 10% overlap with DECIPHER regions underwent manual review to identify any CNVs with at least 80% overlap with previously identified pathogenic CNVs in internal databases and were flagged as putatively pathogenic.

In total, 206 putatively pathogenic CNVs (0.56% of 36,631 rare CNVs) were identified. 86 (0.23% of 36,631 rare CNVs) of these CNVs were deletions that contained at least one gene with a haploinsufficiency score of 3. The ClinGen haploinsufficiency score filters were not mutually exclusive as of the 86 deletions containing a gene with a score of 3, 21 also contained a gene with a score of 30. Only 47 (0.13% of 36,631 rare CNVs) deletions contained only one or more genes with a haploinsufficiency score of 30. Five (0.01% of 36,631 rare CNVs) were duplications with a triplosensitivity score of 3. There were also nine deletions, and 59 duplications unique to the DECIPHER dataset, highlighting the need for multiple filters.

In the putative pathogenic dataset, there were six instances where a duplication of *APP*, which is associated with Early-onset Alzheimer disease (EO-AD) with cerebral amyloid angiopathy was identified [42]. Four additional participants with a full or partial duplication of the *APP* gene were also identified for a total of 10 (0.10% of 10,488 participants in the HostSeq cohort). These four duplications were not flagged as putatively pathogenic using our filtering criteria.

## Discussion

As our results demonstrate, integrating CNV analysis into GS pipelines can help understand genomic copy number variation in the general population. We identified the full spectrum of CNVs in the HostSeq cohort. Of the total 8,543,334 CNV calls, only 36,631 (0.42%) calls remained after applying our filtration criteria to identify rare CNVs, 41.65% of which were singletons. There was a lower percentage of rare singleton CNVs in our cohort when excluding the X and Y chromosomes at 41.0% vs the 53.8% in the UKBiobank, likely due to the differences in the minimum CNV size threshold, and cohort size [43].

Looking closely at the secondary filters applied during analysis, our results for the tiered stratification of deletions based on inheritance were comparable to those of Boone et al., with Tier 1 deletions being the most common in both datasets, followed by Tier 3 and lastly Tier 2 [29]. Smaller deletions and deletions encompassing fewer genes were more frequently categorized as Tier 1 and 3. In comparison, larger deletions and deletions encompassing many genes were categorized as Tier 2, as was expected based on the Tier 2 requirements (Fig 3).

With regard to the recurrent pathogenic CNVs in DECIPHER (Table 2), the frequencies of these CNVs in our cohort are comparable to that of the controls presented by Rosenfeld et al. [32]. Most of the regions identified by Rosenfeld et al. were captured for curation using the DECIPHER region filter, except for the *NIPA1* and *SH2B1* CNVs, which were smaller than previously identified pathogenic CNVs. The *SH2B1* deletions were captured for review using the ClinGen dosage filter and underwent manual review, while the *NIPA1* deletions and *SH2B1* duplications did not, suggesting that applying stringent filtering criteria may result in the exclusion of potentially clinically relevant CNVs. All recurrent CNVs identified by Rosenfeld et al. were also identified in the HostSeq cohort, except for the *HNF1B* deletion and the *GJA5* duplication [32]. The prevalence estimates for these recurrent CNVs were within the range of what has been published, with the exception of the *NIPA1* deletions [44]. This is likely due to a difference in filtration criteria, as Smajlagić et al. only considered CNVs spanning at least 50% of the region of interest, and the *NIPA1* CNVs in our cohort had less than 10% overlap [44]. As the *SH2B1* deletions and duplications also had less than 10% overlap, the adjusted prevalence estimates for these 3 CNVs would be 0 (95% C.I.: 0.00, 3.66), which overlaps with the 95% C.I. that have been published [44].

Self-reported medical history was available for only eight of the participants carrying these recurrent pathogenic CNVs, none of whom indicated a personal history of the disease associated with the identified recurrent CNV. The frequency of these recurrent CNVs in a large cohort representative of the general Canadian population provides valuable insight into their incidence. Our results also suggest that these CNVs may exhibit reduced penetrance or variable disease severity, which is crucial information for genetics professionals, such as genetic counselors, when offering risk counseling to patients with these findings.

Of the 36,631 rare CNVs, 1,136 had a ClinGen Dosage Score of 3 or 30, and 728 CNVs had greater than 10% overlap with a DECIPHER region. Additionally, approximately 1.30% (136 participants) carried a deletion of a gene with a haploinsufficiency score of 3. This is higher than previously published estimates of 0.58% from 2020 [45]. This difference is likely explained by ClinGen’s curation of additional dosage sensitive genes since 2020.

The 1,136 ClinGen dosage and 728 DECIPHER CNVs were manually reviewed, and after removal of duplicate calls, 206 CNVs (0.56% of 36,631 rare CNVs) were deemed putatively pathogenic (S8–S11 Tables). We want to emphasize that the estimate of 0.56% putatively pathogenic variants in the rare dataset is not equal to the true number of pathogenic variants in our cohort. Based on our filtration criteria, we were only able to call variants with 80% overlap with previously known pathogenic CNVs as pathogenic. CNVs with less overlap, or no known pathogenic CNVs in the region were excluded. The true number of pathogenic CNVs is likely higher in the HostSeq cohort. For instance, 39 participants carried a deletion of *HBA1* and *HBA2*, which are associated with carrier status for alpha-thalassemia, yet none of these participants had met the filtration criteria to be flagged as putatively pathogenic [5]. Based on the ACMG and ClinGen technical standards for interpretation of copy number variants, the deletions involving *HBA1* and *HBA2*, would be classified as likely pathogenic at a minimum [46]. In fact, most of the 1,136 CNVs with a ClinGen dosage score would meet the criteria to be classified as likely pathogenic if considering overlap with established or predicted haploinsufficient or triplosensitive regions alone, as detailed family history and/or inheritance is not available for these cases. One exception to this would be for CNVs that do not result in nonsense-mediated decay in the case of deletions, or those that are fully contained within benign regions [46].

Furthermore, the estimate of 0.56% putatively pathogenic variants is lower than previously published data from Canadian cohorts which ranged from approximately 2% to 16.1% clinically relevant variants (S13 Table) [13–21]. However, the previous Canadian cohorts focused primarily on affected individuals and their unaffected parents or siblings, or a small number of individuals from the general population (S13 Table) [13–21]. The differences in demographics between the studies can explain the discrepancy between the two estimates, as does the likelihood that the figure of 0.56% likely underestimates the true number of pathogenic variants. Nevertheless, this discordance highlights the need for a dataset representative of the general population to generate more accurate estimations about the prevalence of disease and population frequencies.

Although we were able to detect a wide spectrum of CNVs using the HostSeq data, our analysis has limitations. Batch and sequencing site assessment, orthogonal validation, and formal assessments were not conducted. The stringency of our filters prevented analysis from including CNVs smaller than 15kb. This was to reduce the number of false positives, but it is possible that real variants were missed after filtration. Additionally, ClinGen continually evaluates and updates the dosage sensitivity scores of genes. As such, the number of pathogenic CNVs in our dataset reflects what was known at the time the CNV calling was done in May 2025. Nonetheless, the CNV data collected from the HostSeq cohort is a valuable resource as calls involving any newly updated genes may be present in our dataset and can be used to determine population frequencies and the penetrance of conditions.

## Conclusion

In summary, we detected a wide range of CNVs in the HostSeq cohort and identified rare, clinically significant CNVs after filtration. The HostSeq cohort reflects the general, ostensibly healthy Canadian population, and the frequencies of some rare CNVs have not previously been identified, making the data an invaluable resource in understanding the spectrum of human variation.

## Supporting information

S1 File Prevalence analysis script. (MD)

S1 Table Count of all CNVs. (XLSX)

S2 Table Count of filtered CNVs. (XLSX)

S3 Table Count of rare CNVs. (XLSX)

S4 Table Rare dataset unique calls. (XLSX)

S5 Table Prevalence of CNVs in each gene. (XLSX)

S6 Table OMIM dataset. (XLSX)

S7 Table Tiered stratification. (XLSX)

S8 Table Recurrent neuropsychiatric CNVs. (XLSX)

S9 Table ClinGen Haplo 3 dataset. (XLSX)

S10 Table ClinGen Triplo 3 dataset. (XLSX)

S11 Table ClinGen Haplo 30 dataset. (XLSX)

S12 Table DECIPHER dataset. (XLSX)

S13 Table Canadian literature. (XLSX)
